# Prevalence and Progression of Diabetic Retinopathy in a Tertiary Care Setting: An Initial Review With Recommended Screening Protocols

**DOI:** 10.7759/cureus.69296

**Published:** 2024-09-12

**Authors:** Pir Salim Mahar, Mohammad Daniyal Monis, Muhammad Fahadullah Khan, Shahid Ahsan, M. Saleh Memon

**Affiliations:** 1 Ophthalmology, Isra Postgraduate Institute of Ophthalmology, Karachi, PAK; 2 Ophthalmology, Aga Khan University Hospital, Karachi, PAK; 3 Biochemistry, Jinnah Medical and Dental College, Karachi, PAK

**Keywords:** diabetes mellitus, diabetic macular edema, non-proliferative diabetic retinopathy, proliferative diabetic retinopathy, vision screening

## Abstract

Introduction: The objective of this study is to evaluate diabetic patients with either normal fundus or non-proliferative diabetic retinopathy (NPDR) changes, examine retinal alterations during follow-up, and propose follow-up guidelines within a tertiary eye care setting.

Methods: A five-year prospective longitudinal study is being conducted at the Diabetic Clinic of Al Ibrahim Eye Hospital/Isra Postgraduate Institute of Ophthalmology, Karachi. Induction for the research took place from October 2021 to March 2022, and a two-year preliminary report is presented here. Newly diagnosed type II diabetic patients with normal fundus or NPDR of any stage, irrespective of age, gender, or glycemic status, who were willing to participate and agreed to follow-ups, were included. Patients with proliferative diabetic retinopathy (PDR), diabetic macular edema (DME), fundus non-visibility, or systemic complications of diabetes were excluded.

Results: A total of 251 patients were enrolled, consisting of 80 individuals with a normal fundus and 171 with different stages of NPDR, including mild (N=59), moderate (N=91), and severe (N=21) retinopathy. The incidence of progression from mild to moderate NPDR was noted to be 52.5%, with a median time of 3.5 months. Progression from moderate to severe NPDR occurred in 37.1% of cases, with a median time of 4.5 months. Similarly, DME developed in 5% of patients with mild NPDR over eight months, in 22.2% with moderate NPDR over seven months, and in 37.5% with severe NPDR over 4.4 months.

Conclusion: This study emphasizes the urgent need to revise diabetic retinopathy (DR) monitoring protocols for our Pakistani (Southeast Asian) population. The rapid progression of NPDR and the high rates of DME development demand more frequent screenings. Current guidelines recommending annual screenings are inadequate. Biannual screenings for patients with a normal fundus or mild NPDR, and quarterly assessments for those with moderate or severe NPDR, are necessary.

## Introduction

Pakistan currently ranks third in the world for diabetes prevalence, following China and India. With nearly 33 million individuals affected, this number is projected to soar to 629 million globally by 2045 [1,2]. Approximately one in three adults in Pakistan is estimated to have diabetes, with many being diagnosed with type 2 diabetes during their working years, while others are diagnosed only after complications occur.

As the prevalence of diabetes continues to escalate exponentially, the incidence of diabetic eye complications is expected to follow suit. Diabetic macular edema (DME) and proliferative diabetic retinopathy (PDR) stand out as the most common causes of diabetes-related vision loss. DME arises from vessel leakage leading to edema, while PDR results from retinal ischemia promoting neovascularization and culminating in vitreous hemorrhage, retinal detachment, neovascular glaucoma, and ultimately severe vision loss [3]. Timely detection of the disease allows for its arrest or even regression through proper diabetes management and with anti-VEGF treatment, or laser therapy, thus averting visual impairment [4].

The American Academy of Ophthalmology (AAO) has outlined recommended follow-up intervals, recommending screenings every one to two years for diabetics without any diabetic retinopathy (DR), annually for those with mild NPDR, every six to nine months for moderate non-proliferative diabetic retinopathy (NPDR), and every three to six months for severe NPDR [5]. Indian guidelines classify individuals with no DR and mild NPDR as not requiring referral, recommending referral for individuals with more severe DR (moderate NPDR and above, with or without DME) [6]. Additionally, they emphasize the necessity for diabetes registries to ensure annual DR screenings. However, there are currently no such recommendations in Pakistan from the Pakistan Diabetic Association or the Ophthalmological Society of Pakistan (OSP) regarding retinopathy and DME screening.

The present study seeks to establish screening interval guidelines for early diagnosis, based on the emergence, progression, and regression patterns of retinal changes leading to various DR stages and DME.

This article was previously posted to the Research Square preprint server on June 7, 2024.

## Materials and methods

Study design

This prospective longitudinal study was initiated at the Diabetic Clinic of Al Ibrahim Eye Hospital/Isra Postgraduate Institute of Ophthalmology, Karachi, in October 2021 and is projected to continue until October 2026. A preliminary report spanning two years is presented herein. The sample size was determined using OpenEpi sample software, considering a 28.78% prevalence of DR with a 95% confidence interval [7]. Accordingly, the calculated sample size was 240. Sampling was conducted through convenience sampling methods. Patients were recruited from the diabetic clinic between October 2021 and March 2022 according to the inclusion and exclusion criteria.

The grading of DR stages was performed utilizing the "international clinical diabetic retinopathy and DME disease severity scales" [8]. Inclusion criteria encompassed newly diagnosed type 2 diabetic patients exhibiting a normal fundus or NPDR of any stage, irrespective of age, gender, or glycemic status, who were willing to participate and consented to follow-ups. Exclusion criteria comprised patients with PDR, macular edema, non-visible fundus, or severe complications of diabetes such as renal failure. In the event of any patient developing vision-threatening diabetic retinopathy (VTDR) during the study period, necessary treatment was promptly initiated.

Patients underwent a comprehensive examination, which included history-taking, visual acuity assessment using Snellen’s chart, and fundus examination conducted by an optometrist using a non-mydriatic fundus camera (Canon CR2). Subsequently, measurements of blood pressure (BP), weight, height, and random blood sugar (RBS) were taken. The status of diabetes control was evaluated by a diabetologist. The grading of DR was performed by a senior ophthalmologist and a medical retina consultant utilizing slit-lamp 90D biomicroscopy. Detailed proformas were completed for each patient. Additionally, all patients underwent biochemical tests such as glycated hemoglobin (HbA1c), lipid profile, and serum creatinine. The test reports were collected and entered into the system by a counselor and subsequently shared with the patients. If any changes in the treatment regimen were deemed necessary, individuals were scheduled to visit the diabetic clinic on a date convenient for both the patient and the diabetologist.

The data of study patients were stratified into four groups: those who progressed, those who remained stable, those who regressed, and those who developed DME. These groups were then analyzed with respect to various biochemical parameters.

In consideration of ethical concerns, the research team implemented an incentive program. Each participant in the study received free diabetic medications equivalent to three months' worth, along with transport allowances. This initiative significantly reduced dropouts from the study.

Ethical approval and statistical analysis

Prior approval was obtained from the hospital’s Research Ethics Committee, with the study protocol assigned the number REC/IPIO/2021/040-A. All principles of the Declaration of Helsinki were followed in compliance with the regulations of the professional code for physicians. The data were analyzed using the Statistical Package for the Social Sciences (SPSS) version 22.0. Mean±standard deviation (SD) was calculated for parametric data. After assessing normality through the Shapiro-Wilk test, the data for best-corrected visual acuity (BCVA) in logMAR were found to be non-parametric [9]. Therefore, the median and interquartile range (IQR) were computed for BCVA. The Kruskal-Wallis test was employed to compare BCVA among the different groups [10]. Additionally, the Chi-square test was utilized to examine the association between retinopathy stages and the duration of diabetes, a confidence interval of 95% was used, and p<0.05 was considered significant. For the comparison of means of biochemical parameters among groups, one-way ANOVA was performed followed by post-hoc Tukey’s test for significance between the means of each group as expressed for the different groups with a 95% confidence interval and p<0.05 was considered significant.

## Results

A total of 251 patients were enrolled in this study after taking informed consent. There were not reported instances of withdrawal of consent of participation. The 251 participants comprised 80 (31.9%) with a normal fundus, 59 (23.5%) with mild NPDR, 91 (36.3%) with moderate NPDR, and 21 (8.4%) with severe NPDR. Baseline characteristics and biochemical parameters of these patients are detailed in Table 1.

**Table 1:** Baseline characteristics and biochemical parameters of patients The data has been represented as the number of participants and the corresponding percentage (N (%)) and mean±SD with p-value (p<0.05 considered significant). HDL, high-density lipoproteins; LDL, low-density lipoproteins; TRIG, triglycerides

Descriptive	Normal fundus, N=80 (31.9%)	Mild NPDR, N=59 (23.5%)	Moderate NPDR, N=91 (36.3%)	Severe NPDR, N=21 (8.4%)	P-value
Age (yrs) (mean±SD)	49.2±9.36	50.98±8.28	52.10±7.58	52.01±8.04	0.289
Male/female, N (%)	33 (41.2)/47 (58.2)	29 (49.1)/30 (50.9)	55 (60.4)/36 (39.6)	13 (62)/8 (38)	0.021
Duration of diabetes, N (%)					
≤5 yrs	44 (56.4)	14 (17.9)	13 (16.6)	7 (8.9)	0.001
6-10 yrs	20 (25)	26 (32.5)	30 (37.5)	4 (5)	
>10 yrs	16 (19)	19 (22.6)	48 (57.1)	10 (11.9)	
Treatment, N (%)					
Oral	55 (34.3)	40 (25)	51 (31.8)	14 (8.7)	0.003
Insulin	3 (11.1)	6 (22.2)	16 (59.2)	2 (7.4)	
Diet	10 (52.6)	5 (26.3)	3 (15.7)	1 (5.2)	
Combination	12 (26.6)	8 (17.7)	21 (46.6)	4 (8.8)	
Biochemical history (Mean±SD)					
RBS	216.67±79.2	283.80±104.40	308.13±109.76	279.21±88.02	0.027
HbA1c	10.05±4.98	9.64±2.61	9.93±2.22	10.61±1.97	0.311
Serum creatinine	0.93±0.18	0.98±0.94	0.94±0.28	0.96±0.18	0.452
HDL	35.67±6.72	36.45±8.94	34.68±8.70	39.36±6.40	0.189
LDL	129.04±27.35	111.41±29.31	128.85±41.73	112.90±32.27	0.037
TRIG	182.85±96.8	196.35±97.16	197.21±121.67	156.81±65.45	0.041

**Figure 1:**
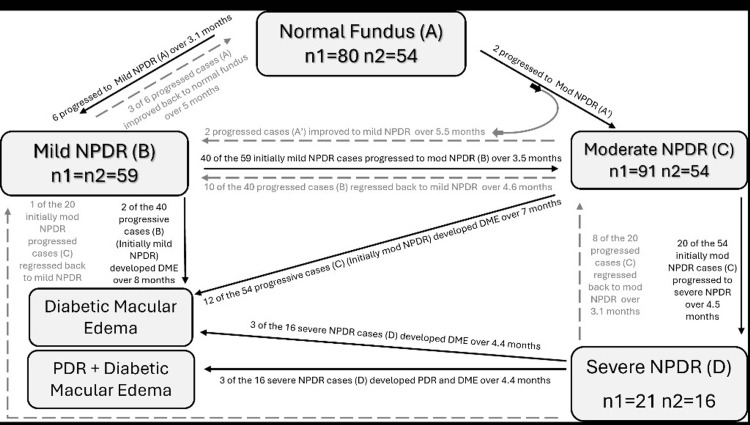
Flowchart for progression, regression, and DME Arrows show a change in category. Black line and black label, progression; grey line and grey label, regression; n1, number of patients at time of induction in study; n2, number of patients completing two year follow-up; (A)-(D), labels for each group indicator; DME: diabetic macular edema

Out of the 80 patients initially diagnosed with a normal eye fundus, 26 (32.5%) did not return for follow-up appointments. Of the 54 patients who continued with the study (67.5%), the majority, 46 individuals (85.2%), maintained a stable fundus over a two-year period. However, among these, patients with initially normal fundus, eight patients (14.8%) showed progression to NPDR (Figure 1).

Within these eight cases of progression to NPDR, six patients (11.1%) developed mild NPDR, while two patients (3.7%) progressed to moderate NPDR. In these patients, on average, NPDR first appeared around 3.1 months after the initial diagnosis. Interestingly, three of the six patients who had progressed to mild NPDR saw their condition improve, returning to a normal fundus after about five months on average. Similarly, the two patients who had progressed from normal fundus at presentation to moderate NPDR experienced a regression to mild NPDR within approximately 5.5 months. Throughout the study, none of the patients developed DME. Additionally, we found a moderate positive correlation between stable and progressive cases when looking at patients' HbA1c levels though this was not statistically significant (r=0.424, p>0.05).

Among the 59 patients with mild NPDR at presentation, 40 (67.7%) progressed to moderate NPDR, with a probability of 52.5% (N=21). The median time to the first incidence of moderate NPDR was 3.5 months. The likelihood of developing DME was 5% (two out of 40) over a median time of eight months. Among the 40 progressive cases, 10 cases regressed back to mild NPDR within an estimated duration of 4.6 months and an overall probability of 25%. A weak positive correlation was found between the HbA1c levels of mild NPDR patients and progression to the next stage (r=0.064, p>0.05).

Ninety-one patients diagnosed with moderate NPDR were monitored for fundus changes. Among the 54 (59.3%) cases that completed follow-ups, the probability of progression to severe NPDR from moderate NPDR was 37.1% (20 out of 54), with a median time to the first incidence of severe NPDR of 4.5 months. Among the 20 progressive cases, nine (45%) regressed, eight (40%) to moderate NPDR with a median duration of 3.1 months, and one (5%) to mild NPDR. Similarly, the probability of progression to DME was 22.2% (12 out of 54), with a median time to the first incidence of DME being seven months. A weak correlation was found between the HbA1c levels of moderate NPDR patients and progression to the next stage (r=0.191, p>0.05).

Twenty-one patients diagnosed with severe NPDR were monitored for fundus changes. Among the 16 (76%) cases observed, five (31.3%) remained stable, while three (18.8%) progressed to DME, and three (18.8%) to DME with PDR. The probability of progression to DME was 37.5% (six out of 16), with a median time to the first incidence of DME of 4.4 months. A moderate correlation was observed between the HbA1c levels of severe NPDR patients and progression to the next stage (r=0.572, p>0.05).

These findings underscore the importance of regular monitoring and management of DR, with particular attention to glycemic control as indicated by HbA1c levels. Duration of diabetes exhibited a significant association with different stages (p<0.05), as indicated in Table 2.

**Table 2:** Association of duration of diabetes with different stages The data has been represented as the number of participants, the corresponding percentage (N (%)), and the p-value (p<0.05 considered significant). DME, diabetic macular edema

Stages	Duration of diabetes, N (%)			P-value
	1-5 yrs	6-10 yrs	>10 yrs	
Progression	10 (26.3)	13 (34.2)	15 (39.5)	0.037
Stable	29 (35.8)	34 (42)	18 (22.2)	
Regression	16 (44.4)	7 (19.4)	13 (36.1)	
Developed DME	1 (8.3)	5 (41.7)	6 (50)	

In addition, significant results were observed for systolic BP, RBS, serum creatinine, and HDL levels at different stages (p<0.05). Detailed findings are presented in Table 3.

**Table 3:** Comparison between different parameters among groups The data has been represented as the number of participants, N, and mean±SD with p-value (p<0.05 considered significant). P, progression; R, regression; S, stationary; DME, diabetic macular edema; RBS, random blood sugar; HDL, high-density lipoproteins; LDL, low-density lipoproteins; BP, blood pressure

Systolic BP (N=166)					Diastolic BP (N=166)				
Stage	One way ANOVA (mean±SD)	Stage	Post-hoc Tukey’s test (mean±SD)	P-value	Stage	One way ANOVA (mean±SD)	Stage	Post-hoc Tukey’s test (mean±SD)	P-value
P	131.84±17.5	R	129.03±13.82	0.434	P	82.97±7.14	R	81.81±9.42	0.746
		S	124.20±14.39	0.013			S	80.86±6.55	0.306
		DME	137.50±19.60	0.27			DME	82.50±7.53	0.958
R	129.03±13.82	S	124.20±14.39	0.12	R	81.81±9.42	S	80.86±6.55	0.529
		DME	137.50±19.60	0.102			DME	82.50±7.53	0.78
S	124.20±14.39	DME	137.50±19.60	0.006	S	80.86±6.55	DME	82.50±7.53	0.479
RBS (N=166)					HbA1c (N=124)				
Stage	One way-ANOVA (mean±SD)	Stage	Post-hoc Tukey’s test (mean±SD)	P-value	Stage	One way-ANOVA (mean±SD)	Stage	Post-hoc Tukey’s test (mean±SD)	P-value
P	274.50±79.95	R	226.33±85.10	0.017	P	9.17±1.99	R	7.80±1.14	0.541
		S	240.65±87.45	0.047			S	9.90±4.42	0.641
		DME	297.58±98.10	0.419			DME	9.66±2.15	0.853
R	226.33±85.10	S	240.65±87.45	0.407	R	7.80±1.14	S	9.90±4.42	0.305
		DD	297.58±98.10	0.014			DME	9.66±2.15	0.525
S	240.65±87.45	DME	297.58±98.10	0.034	S	80.86±6.55	DME	9.66±2.15	0.921
Serum creatinine (N=105)					HDL (N=105)				
Stage	One way-ANOVA (mean±SD)	Stage	Post-hoc Tukey’s test (mean±SD)	P-value	Stage	One way-ANOVA (mean±SD)	Stage	Post-hoc Tukey’s test (mean±SD)	P-value
P	1.04±0.53	R	0.95±0.23	0.545	P	44.66±27.18	R	36.10±8.56	0.192
		S	0.93±0.24	0.232			S	35.53±11.30	0.028
		DME	1.25±0.96	0.23			DME	45.12±23.76	0.949
R	0.95±0.23	S	0.93±0.24	0.878	R	36.10±8.56	S	35.53±11.30	0.925
		DME	1.25±0.96	0.137			DME	45.12±23.76	0.283
S	0.93±0.24	DME	1.25±0.96	0.045	S	35.53±11.30	DME	45.12±23.76	0.031
LDL (N=105)					Triglycerides (N=105)				
Stage	One way-ANOVA (mean±SD)	Stage	Post-hoc Tukey’s test (mean±SD)	P-value	Stage	One way-ANOVA (mean±SD)	Stage	Post-hoc Tukey’s test (mean±SD)	P-value
P	114.11±42.34	R	108.90±25.75	0.743	P	208.85±174.15	R	213.10±109.82	0.926
		S	124.61±43.91	0.292			S	188.70±97.36	0.474
		DME	125.50±51.88	0.51			DME	199.87±54.56	0.854
R	108.90±25.75	S	124.61±43.91	0.285	R	213.10±109.82	S	188.70±97.36	0.558
		DME	125.50±51.88	0.416			DME	199.87±54.56	0.819
S	124.61±43.91	DME	125.50±51.88	0.956	S	188.70±97.36	DME	199.87±54.56	0.807

Visual acuity was also compared across the four groups. A Kruskal-Wallis H test revealed a significant difference in BCVA among the four groups, χ2 (3)=23.527, p=0.001. The median (IQR) BCVA was 0.3 (0.18) logMAR for the progression group, 0.2 (0.10) logMAR for the stable group, 0.2 (0.10) logMAR for the regression group, and 0.45 (2.20) logMAR for the group that developed DME.

These findings underscore the importance of considering various clinical parameters, including duration of diabetes, BP, blood sugar levels, renal function, lipid profile, and visual acuity, in the management and prognosis of DR.

## Discussion

In this study, the likelihood of DR development in individuals with normal fundi was 14.8% within a median time of 3.1 months. The probability of progressing from mild NPDR to moderate NPDR was 52.5% and from moderate NPDR to severe NPDR was 37.1%, with median durations of 3.5 and 4.5 months, respectively. Additionally, the likelihood of developing DME in mild, moderate, and severe NPDR cases was 5%, 22.2%, and 37.7%, respectively, with median durations of 8, 7, and 4.4 months over a span of two years.

This indicates that if diabetics with normal fundus or mild NPDR are scheduled for follow-up after one year or longer, as suggested by the AAO and Indian guidelines, there is a risk of missing the early diagnosis of mild NPDR in 14% of normal fundus cases, progression to moderate NPDR in 26%, and development of DME in 5% of mild NPDR cases [5,6]. Therefore, it is advisable for diabetics with normal fundus and mild NPDR to undergo screening every six months instead of waiting for one year after the initial screening.

The follow-up interval for moderate and severe NPDR is nearly in line with the AAO guidelines, which recommend six to nine months for moderate NPDR and three to six months for severe NPDR [5]. However, it would be prudent to err on the side of caution by reducing the interval to three to six months for moderate NPDR and three to four months for severe NPDR.

Moshfeghi and colleagues, looking at patterns of DR progression in USA Clinical Practice, demonstrated that over a five-year period, the likelihood of progression from mild and moderate NPDR to severe NPDR was 5.8% and 17.6%, respectively, compared to 22.2% and 37.7% in our study [11]. The higher percentages observed in our study may be attributed to inadequate management of diabetes in our region or potentially influenced by genetic or ethnic factors, as suggested by studies from European countries. These studies have indicated an increased susceptibility of Asian populations to diabetes and DR. For instance, the prevalence of any DR was 37.4% in white Europeans compared to 42.35% in South Asians. Similarly, the prevalence of VTDR was 10.3% in South Asians compared to 5.5% in white Europeans [12].

In US Clinical Practice, the incidence of DME was reported to be 44.6% and 62.6% in moderate and severe NPDR, respectively, over a five-year period [11]. In comparison, our present study found that 22.2% of patients with moderate NPDR and 37.8% with severe NPDR developed DME within two years. Given the difference in follow-up duration, it is plausible that within the next three years, we may observe similar figures.

The development, progression, and regression of DR/DME depend on both modifiable and non-modifiable risk factors. Modifiable risk factors for DR include chronic hyperglycemia, nephropathy, hypertension, and dyslipidemia. This study reveals significant differences among systolic BP levels in progressing and regressing cases, as well as in stable and developed DME cases (p<0.05). A similar relationship was demonstrated in a study by Liu and co-workers from Singapore, where DR progression was associated with high systolic BP [13]. Regarding hyperglycemia, there were significant differences among RBS levels in progressive and regressive cases, as well as in progressive and stable cases (p<0.05). However, no significant difference was found in any group with respect to HbA1c levels. A retrospective cohort study by Kim and colleagues found no significant differences in the proportion of progression to moderate NPDR or worse DR between individuals with high and low HbA1c levels [14]. Interestingly, HbA1c is more relevant to diabetes and coronary disease than to retinopathy [15]. It has also been reported that HbA1c is related to the prevalence of diabetic nephropathy but not to retinopathy [16]. In another study by Foo and co-workers on Asian subjects with type 2 diabetes, it was found that HbA1c variability was not associated with the presence of moderate DR [17]. Significant differences in serum creatinine and high-density lipoproteins (HDL) levels (p<0.05) were observed between stable fundus and developed DME cases.

Another non-modifiable factor is the duration of diabetes; DR tends to become more progressive as the duration of diabetes increases. Our study indicates that 26.3%, 34.2%, and 39.5% of individuals in the progressive group had a duration of diabetes of one to five years, six to 10 years, and over 10 years, respectively. Similarly, DME also appears more frequently as the duration of diabetes increases. The study reveals that 8.3%, 41.7%, and 50% of individuals who developed DME had a duration of diabetes of one to five years, six to 10 years, and over 10 years, respectively (p<0.05). Shrote and Diagavane in their work on Indian subjects demonstrated a significant association (p=0.04) between the duration of diabetes and the severity of DR [18]. A similar relationship has been reported by Niazi and colleagues from Pakistan as well (p<0.05) [19].

The present study is notable for its observation of early development of DR as well as DME. The occurrence of DME in the first group (one to five years) is unexpected. These changes manifest within a relatively short time frame of 3.1 to 4.5 months, even among individuals with less than five years of disease duration. The early emergence of microvascular changes can be attributed to the late diagnosis of the disease. In rural communities, individuals with diabetes often lack awareness of their condition, leading to delayed detection [20].

Given the observed accelerated progression of NPDR and the development of DME, it is recommended to shorten the referral interval for subsequent screenings. Specifically, intervals of six months for individuals without DR and three to six months for all NPDR cases are advised. To facilitate early follow-up and mitigate the risk of diabetes-related blindness, policymakers, healthcare providers, and the community should collaborate to improve health education, raise awareness, and enhance the affordability of healthcare services.

In essence, this study emphasizes the importance of regular screening, tailored follow-up intervals, and comprehensive management strategies to mitigate the progression of DR and its associated complications. Additionally, efforts to raise awareness and improve access to healthcare services, particularly in underserved populations, are crucial in addressing the early onset and rapid progression of diabetic eye disease.

This study is limited by its single-center, tertiary-based design. Future research would benefit from community-based, multicenter studies to enhance generalizability. Additionally, the cost-effectiveness of frequent screenings should be explored. Fasting blood sugar (FBS) measurements were omitted due to logistical challenges faced by patients traveling long distances and experiencing lengthy waiting periods in hospitals.

## Conclusions

The evidence from this study unequivocally demonstrates the urgent necessity for revising current DR monitoring protocols for our Pakistani (Southeast Asian) population. The rapid progression of NPDR and the alarming development rates of DME within shorter durations demand immediate action. Current international guidelines recommending annual screenings are grossly inadequate and risk severe vision loss among diabetic patients in our population. It is imperative that we implement more aggressive monitoring protocols: biannual screenings for patients with a normal fundus or mild NPDR and quarterly assessments for those with moderate or severe NPDR.

Furthermore, healthcare systems must prioritize education and access to ensure adherence to these stringent follow-up schedules. Without these critical changes, we will continue to face preventable cases of blindness due to diabetic complications. As a nation, the time to act is now, and the medical community must lead the charge in overhauling current international protocols for more population-tailored ones. By doing so, we can significantly reduce the incidence of severe vision impairment and improve the quality of life for millions of diabetic patients locally and beyond.
